# Activation of Transposable Elements in Human Skeletal Muscle Fibers upon Statin Treatment

**DOI:** 10.3390/ijms24010244

**Published:** 2022-12-23

**Authors:** Braulio Valdebenito-Maturana, Franco Valdebenito-Maturana, Mónica Carrasco, Juan Carlos Tapia, Alejandro Maureira

**Affiliations:** 1 Instituto de Investigación Interdisciplinaria, Vicerrectoría Académica, Universidad de Talca, Campus Talca, Talca 3460000, Chile; 2 Centro de Investigación en Células Madres y Neurociencias, Universidad de Talca, Campus Talca, Talca 3460000, Chile; 3 Escuela de Medicina, Universidad de Talca, Campus Talca, Talca 3460000, Chile

**Keywords:** transposable elements, gene regulation, statins, pharmacogenomics, cholesterol, cardiovascular diseases

## Abstract

High cholesterol levels have been linked to a high risk of cardiovascular diseases, and preventative pharmacological care to lower cholesterol levels is critically important. Statins, which are hydroxymethylglutaryl-coenzyme A (HMG-CoA) reductase inhibitors, are drugs used to reduce the endogenous cholesterol synthesis, thus minimizing its pathophysiological effects. Despite the proven benefits, statins therapy is known to cause a number of skeletal muscle disorders, including myalgia, myopathy and myositis. The mechanisms underlying such statin-induced side effects are unknown. Recently, a group of genes and molecular pathways has been described to participate in statin-induced myopathy, caused by either simvastatin or rosuvastatin, although the mechanism by which changes in gene regulation occur was not studied. Transposable Elements (TEs), repetitive elements that move within the genome, are known to play regulatory roles in gene expression; however, their role in statin-induced muscle damage has not been studied. We analyzed the expression of TEs in human skeletal fiber cells treated with either simvastatin or rosuvastatin, as well as their respective controls, and identified TEs that change their expression in response to the treatment. We found that simvastatin resulted in >1000 differentially expressed (DE) TEs, whereas rosuvastatin resulted in only 27 DE TEs. Using network analysis tools, we predicted the impact of the DE TEs on the expression of genes and found that amongst the genes potentially modulated by TEs, there are some previously associated to statin-linked myopathy pathways (e.g., AKT3). Overall, our results indicate that TEs may be a key player in the statin-induced muscle side effects.

## 1. Introduction

Cardiovascular diseases are the most prevalent causes of death in developed countries [1]. High levels of low-density lipoprotein (LDL) cholesterol have been singled out as a key culprit. This accumulates on the walls of blood vessels, promoting atherosclerosis and increasing the risk of cardiovascular diseases such as coronary heart attack, stroke, etc. [2]. Thus, high LDL cholesterol levels are life-threatening, and most healthcare providers attempt to regulate its blood levels through pharmacological approaches [3]. The hydroxymethylglutaryl-coenzyme A (HMG-CoA) reductase inhibitors known as statins have been one of the most important drugs used to target LDL cholesterol levels [4]. Statin are classified by their chemical origin (natural or synthetic) and by their structure into two classes (Type I and Type II). Depending on their lateral group, statins range from very hydrophobic (e.g., simvastatin) to hydrophilic (e.g., rosuvastatin) [5,6]. Hydrophobic statins can impact a greater range of cells and tissues, while hydrophilic statins tend to be liver-specific [7]. Regardless of the type used, between 40–60% of statin treatment discontinuation cases were associated with statin-associated muscle symptoms (SAMS), including widespread skeletal muscle side effects, often with muscle inflammation (myositis), muscle pain (myalgia) and muscle weakness (myopathy) [5]. Several hypotheses, which were proposed to explain the mechanisms underlying SAMS, have been tested, including Vitamin D deficiency, statin-mediated mitochondrial disfunction, and atrophy induced by statin activation of the phosphoinositide 3-kinase (PI3K)/Akt pathway [8]. A recent study compared the gene expression in statin-treated versus control human skeletal fiber cells from 22 cell populations [5]. These genetic analyses revealed changes in regulatory processes, such as RNA synthesis and processing and lipid metabolism. Along with cholesterol metabolism, two other pathways are altered by statin treatment: mitochondrial beta oxidation and eicosanoid synthesis. Defects in mitochondrial beta oxidation have been linked to both skeletal and cardiac myopathies [9], while alterations in eicosanoid synthesis result in increased cellular inflammation [10], supporting the notion that statins lead to inflammation and a metabolic stress state. The change in expression of many genes from the same pathway, and from pathways of the same cellular processes, suggest an interplay of gene regulatory elements in the statin-induced effect on human skeletal muscle fibers. 

Transposable Elements (TEs) are genetic elements with the ability to replicate and transpose (move) within the genome of germline and specific somatic cells [11,12]. Depending on the transposition intermediates used, TEs are classified into two major classes: retrotransposons (class I) and DNA transposons (class II). Retrotransposons perform reverse transcription on their RNAs, resulting in new copies of themselves, which are later inserted in another genomic location. TEs from this class are further subdivided into Long Terminal Repeats (LTRs), Long Interspersed Nuclear Elements (LINEs) and Short Interspersed Nuclear Elements (SINEs). In contrast, DNA transposons encode proteins that allow excision from the genome locus and its insertion elsewhere. Most TEs are unable to transpose as a result of the accumulation of inactivating mutations, and, based on their divergence rate, they can also be classified into “young” or “old” TEs [13,14]. In addition to inactivating mutations, host genomes have several mechanisms to regulate TE expression at the transcriptional and post-transcriptional level, such as DNA methylation, chromatin modifications, RNA interference pathways and through activity of Krüppel-associated box (KRAB) zinc-finger proteins, amongst others (reviewed in [15,16]). Most TEs are silenced by DNA methylations [17], with this modification being commonly found on LINE and SINE TEs [15]. Interestingly, there is evidence linking differential methylation of TEs with changes in neighboring gene expression [17]. A classic example of this is the de-methylation of a TE upstream of the *agouti* gene, which causes this TE to act as an alternative transcriptional start site and become spliced into the gene [18]. This event leads mice to have yellow fur and become obese [16,17,18].

It is now well-accepted that TE expression is linked to gene expression in several ways. It has been suggested that the contribution of TE-derived gene regulatory elements might have played a role in vertebrate adaptation, and for humans, it is currently estimated that 20% of regulatory elements are derived from TEs [15]. After bursts of activity and mobilization, TEs can become fixed in genomes (“domesticated”), due to the fact that they carry cis-regulatory genic elements, such as promoters and Transcription Factor Binding Sites (TFBSs), effectively participating in the formation of gene regulatory networks [15,19,20]. In this regard, it has been hypothesized that TEs drove the evolution of KRAB zinc-finger proteins, with implications in modulating gene expression [21]. Indeed, there is evidence pointing that knockouts of KRAB-ZFPs or their associated proteins cause modifications in TE expression as well as that of neighboring genes. For example, knockout of the KRAB ZFP809 resulted in activation of genes near LTRs that are normally repressed by that protein, and knockout of the KAP1 (a co-repressor of the KRAB-ZFPs) caused activation of some LTR TEs and genes near them (reviewed in [21]). A recent work pointed to a more complex relationship between human endogenous retrovirus (HERVs), a type of LTR TEs, and KRAB-ZFPs. Particularly, it was reported that in several types of cancer, upon de-repression of HERVs, KRAB-ZFPs were activated, indicating a negative feedback loop between them [22]. All of the above helps to explain the “evolutionary arms race,” in which old TEs may not have a negative selective pressure against them, and provide the genome with elements that either promote gene expression and/or silence full-length instances [16] (which may correspond to young and actively transposing TEs).

The landmark studies of Barbara MacClintock set the hypothesis for TEs influencing adjacent gene expression, even independent of transposition events of these elements. Thus, the transcriptional regulatory effect of TEs could transition between on and off states [14]. As mentioned earlier, TEs can contain promoter regions, which are able to efficiently recruit RNA polymerases and are responsible for TEs–mRNA expression [23]. In turn, these mRNAs can have important regulatory roles, such as HPAT5, an *Alu*–bearing long intergenic noncoding RNA (lincRNA), which regulates primates’ early embryonic events [24]. Other examples of TE expression associated with gene expression have been reported in mammalian development [25] and some neurodegenerative diseases [13,26]. Different types of stresses can also modify TE expression. Current evidence points to an “epi-transposon hypothesis”, in which TEs can become activated as a result of an epigenetic breakdown associated to external stress, resulting in genomic innovation [19]. Amongst factors that can mediate these epigenetic changes are temperature changes, air pollution, diet, endocrine disruption, drugs and genotoxic agents [15,19,20]. Perhaps the best example of stress-associated TE activation modulating gene expression has been seen under heat stress. Under this type of perturbation, some SINEs can repress transcription by sequestering RNA polymerase II, while in another work, it was seen that genes near some LTRs become up-regulated. In contrast, there is evidence highlighting that under stress, TEs can also become repressed as means of avoiding genomic damage caused by these elements [27]. Collectively, although several works have highlighted changes in TE expression upon stress, what determines the type of TEs that become differentially expressed, and the direction of that change (either up- or down-regulation), still remains to be discovered [15]. Although it is well-accepted now that TEs can influence genes located nearby in the genome, an impact known as a “position effect variegation” (PEV) [16], this phenomenon has not been extensively studied in works that use RNA-Seq to profile gene expression. This is due to the highly repeated nature of TEs within genomes (they represent ~50% of the human genome) that makes the assessment of their expression at the locus-level difficult. 

The goals of the present work were first to determine whether treatment with statins (simvastatin and rosuvastatin) produces changes in the expression of TEs in human skeletal muscle cells, and second, to examine whether such TEs could modulate gene expression. Using state-of-the-art bioinformatic tools, we identified that TEs were differentially regulated in the statin-treated cells. Interestingly, depending on the hydrophobicity of the statin used, the TEs that were up- or down-regulated varied significantly. We also found a strong statistical correlation between intergenic TEs and a set of well-known genes, some of which play important roles in muscle fiber maintenance and function. In summary, our data indicate that TEs become transcriptionally active in response to statin and may modulate the physiopathology of the skeletal muscle fiber.

## 2. Results

### 2.1. Differential Expression of Transposable Elements (TEs) in Human Skeletal Muscle Fibers Treated with Statins

**Figure 1:**
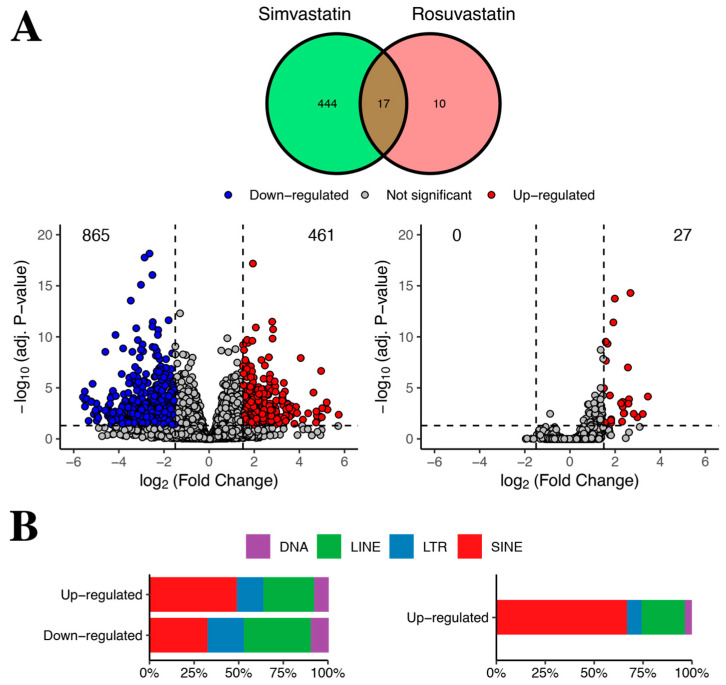
Differential expression of transposable elements in statin-treated human skeletal fibers. (**A**) Venn diagram and volcano plots depicting the expression profile of TEs between left, simvastatin vs. control (vehicle)-treated cells, and right, rosuvastatin vs. control (vehicle)-treated cells. Red circles correspond to up-regulated TEs (increased expression during simvastatin or rosuvastatin treatment relative to control, respectively), whereas blue circles indicate down-regulated TEs. Grey circles show TEs with no statistically significant differences in expression in either condition. The horizontal dashed lines indicate adjusted *p*-value 0.05, whereas the vertical dashed lines indicate −1.5- and 1.5-fold threshold change. Numbers correspond to the TEs which were either up- (red corner) or down- (left blue) regulated for each drug. (**B**) Rectangular boxes show the TE class distribution in percentage (% of the total) among up- and down-regulated TEs for each treatment condition: simvastatin (left) and rosuvastatin (right). Colored squares indicate the color associated with each TE class: purple = DNA, green = LINE, blue = LTR and red = SINE.

Due to technical challenges associated with TE read quantification, they are commonly excluded from RNA-Seq analysis [14]. In the original work by Grunwald et al., they did not analyze TEs nor their role in modulating gene expression. Thus, we decided to explore the expression and potential regulatory activity of TEs using the public RNA–seq dataset from different human skeletal myotube cell cultures exposed to statins. Particularly, to examine whether TE changes occurred in statin-treated cells with respect to controls, we used the SQuIRE tool, which allows locus-specific quantification of TE expression [28]. Then, using DESeq2 [29], we performed differential expression analysis of TEs, revealing that a large number of TEs showed changes in their expression profile upon statin treatment when compared to control cells (Figure 1A,B, for simvastatin and rosuvastatin, respectively).

Interestingly, depending on the statin treatment used, the number of differentially expressed TEs was significantly different. A total of 1326 TEs (up- and down-regulated) changed their expression with the simvastatin treatment, whereas 27 TEs were up-regulated in the rosuvastatin treatment, with no significantly down-regulated elements (Figure 1A). In simvastatin-treated cells, 461 TEs were up-regulated (34.8%), whereas 865 (65.2%) showed reduced expression, with respect to the control (Figure 1A, Supplementary File S1). This is similar to the numbers of differentially expressed genes observed in the original work: 1807 genes were differentially expressed in simvastatin, and only 68 in rosuvastatin. Despite this difference, the authors reported that rosuvastatin can still be associated with myopathies [5]. Collectively, this is in agreement with previous works, indicating that stress can cause either an increase or a decrease in TE expression. In turn, the simvastatin down-regulated TEs could correspond to domesticated instances, which might play a role in normal conditions, as those TEs could be transcribing as a consequence of their host gene transcription (“co-transcription”) [14]. For instance, almost 70% of down-regulated TEs are inside genes.

In terms of TE distribution, we found that in all cases, there was a predominance of SINE TEs followed by LINE TEs (Figure 1B). Notably, the SINE TEs are known to negatively influence mRNA associated processes such as export from the cytoplasm and translation (see discussion). When looking at TEs at the family level, the top three most represented are Alu, L1 and MIR (Supplementary Files S2 and S3 and Supplementary Figure S1), further supporting the notion that both statins have similarities, despite the different number of detected TEs.

### 2.2. Transposable Elements Are Significantly Associated to Genes

**Figure 2:**
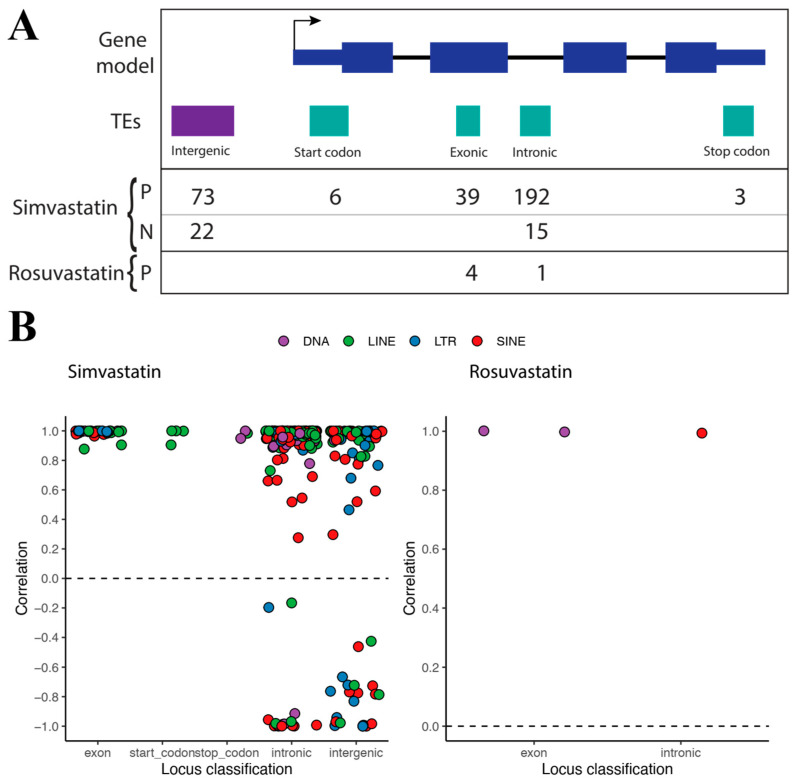
Gene–TE correlation distribution plots according to TE classes. (**A**) Schematics of TE classification based on their location relative to a gene. Numbers below indicate the amount of TEs having positive (“P”) or negative (“N”) correlations, in each treatment (“Simvastatin” or “Rosuvastatin”). (**B**) Correlations distribution plots amongst simvastatin gene–TE pairs (left), and amongst rosuvastatin gene–TE pairs (right). The dashed line at 0 was added to aid in the visual separation of positive and negative correlations.

After the identification of differentially expressed TEs, we aimed to predict their potential role in gene regulation. In order to do so, we employed a rigorous statistical approach (see Methods). First, the TEffectR tool was used to identify genes whose expression could be explained by changes in TE expression. TEffectR applies a linear modeling approach, in which gene expression is modeled as a function of TE expression. To this end, it requires gene-TE pairs and their corresponding expression as inputs. We associated TEs to their closest neighboring genes. Although this is a first step towards understanding gene–TE interactions, a caveat is that it does not prove a causal effect [30]. Afterwards, gene–TE pairs which had statistical significance, according to TEffectR (*p* ≤ 0.05), were kept. For the statistically significant gene–TE pairs, we then calculated their pairwise correlations and plotted them according to the statin used (Figure 2). This was carried out as a means to increase our understanding of the potential gene–TE interactions. In biological terms, the TEffectR approach, followed by correlation calculations, allow us to find the potential gene–TE interactions, and subsequently classify them. A positive correlation obtained in this way might be associated with events in which the TE drives gene expression, or vice versa, whereas negative correlations could be indicative of events in which activation of the TE causes silencing of the gene, or activation of the gene causes silencing of the TE.

For simvastatin, we found a total of 350 statistically significant TE–gene pairs, with 313 (89.4%) of them having positive correlations, and 37 (10.6%) having negative correlations (Figure 2). For rosuvastatin, we only found five statistically significant TE-gene pairs, with all of them having positive correlations (Figure 2). Most of the positive correlations identified in both treatments correspond to TEs located within genes (Figure 2A); there were four in common between rosuvastatin and simvastatin. This suggests that these TEs could be expressed as a consequence of the host gene transcription. This event, known as co-transcription, is not well understood [14]. The mechanisms and their impact on gene regulation remain unclear, although preliminary evidence establishes a link with long non-coding RNAs and enhancer RNAs [14]. Together, this result could point to another role of TEs common for both statins, which may be linked to co-transcription. Interestingly, the intronic TE found for rosuvastatin was amongst the four in common with simvastatin. This TE, AluSq2, located at chromosome 543297558–43297863, is inside the HMGCS1 gene (“*3-hydroxy-3-methylglutaryl-CoA synthase 1*”). HMGCS1 has been previously studied and is reported to have increased expression upon statin treatment [5,31]. Thus, we speculate that it is likely for the AluSq2 TE to mediate HMGCS1 over-expression as a response to statins. For simvastatin, we also found positive correlation values between intergenic TEs and their closest genes, suggesting that these TEs can act as alternative transcription start or termination sites, or as enhancers, depending on the genomic distance with their respective genes [32]. On the other hand, there were several intronic and intergenic TEs that correlated negatively with the associated genes (Figure 2), suggesting that they could potentially be negative regulators of gene expression, and thus play a significant role in the statins-induced skeletal fiber side effects. 

Another caveat to our conservative approach of proposing gene–TE pairs is that in the case of intergenic TEs, we are potentially missing some interactions, because they might not be necessarily associated with their closest gene. Although only 11% of rosuvastatin DE TEs are intergenic, we cannot discard that they might be regulating genes other than those that are located the closest to them. In order to further examine the impact of gene–TE associations, we devised a bioinformatic analysis to identify the genes targeted, as well as the putative pathways involved.

### 2.3. Transposable Elements Might Influence the Expression of Key Genes upon Statin Treatment

As mentioned above, rosuvastatin-treated samples revealed a lower number of gene–TE associations, and all of them showed positive correlations. For simvastatin DE TEs, we also noted a high proportion of positive correlation between the associations with genes. Even though these putative co-transcription events might influence gene regulation, to the best of our knowledge, there is no extensive evidence to support this. For this reason, we continued the analysis with the TEs having negative correlations, which might be more suggestive of regulatory events. This only left us with DE TEs associated with genes under the simvastatin treatment. Overall, we found 36 genes negatively correlated with TEs in response to this very hydrophobic statin: 15 genes associated with intronic TEs and 21 with intergenic TEs (Supplementary File S1). 

Amongst all TEs–gene pairs with negative correlations, we highlighted ZNF556 (zinc finger protein 556), TAOK3 (TAO kinase 3), AKT3 (AKT serine/threonine kinase 3) and SLMAP. The zinc finger proteins are known to play important roles in cellular processes, such as transcriptional regulation, across a range of tissues, including muscle [33]. The TAO kinase 3 has been linked to MAPK14, a molecular pathway activated by cellular stress [34]. The downregulation of AKT3 has been previously associated with mouse muscle fiber maturation in vitro [35], and this AKT3–downregulation can also be associated with metabolic stress in muscle fibers, because the inhibition of AKT signaling pathway (AKT dephosphorylation) by simvastatin decreases glucose uptake [36].

Another relevant gene found to be potentially regulated by TE was SLMAP (sarcolemma associated protein). This gene has a role in myoblast fusion (fiber maturation), a key process for skeletal muscle development and repair [37,38]. 

**Figure 3:**
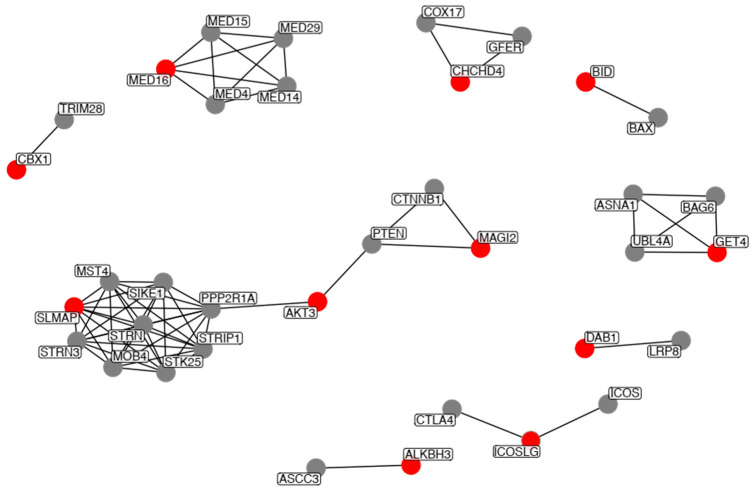
STRING network analysis of the genes potentially modulated by TEs. Input genes are highlighted in red, whereas interacting partners reported by STRING are in grey. Only direct interactions to each of our query genes are shown.

We then explored the protein interacting network of the genes to be potentially be modulated by TEs. To this end, we used the STRING database (Methods). Of all genes, only 11 showed high confidence interactions (STRING confidence value > 0.9), and thus do not appear in the network. The network with the high-confidence interactions is shown in Figure 3.

Most genes comprised individual small networks (<5 interacting partners), with the exception of SLMAP, AKT3 and MAGI2, which comprised a larger network. Of these, SLMAP has the highest number of interacting partners (eight in total), further highlighting the importance of this gene. Amongst all of the interacting partners, we placed emphasis on PPP2R1A, which has direct high-confidence interactions with SLMAP and AKT3 (Figure 3). PTEN, AKT3 and PPP2R1A are part of the PI3K-AKT-mTOR signaling pathway. It has been shown that the muscle-specific knockout mice of several mTOR complex coding genes develop severe myopathy, and that a decrease in mTOR pathway activity is associated to age-related sarcopenia [39], further highlighting the importance of these genes. Moreover, PPP2R1A is also part of the TGF-beta signaling pathway. Activation of the pathway has been implicated in skeletal muscle myopathies. Particularly, the TGF-beta pathway has been reported to be up-regulated in myopathies [40]. 

**Figure 4:**
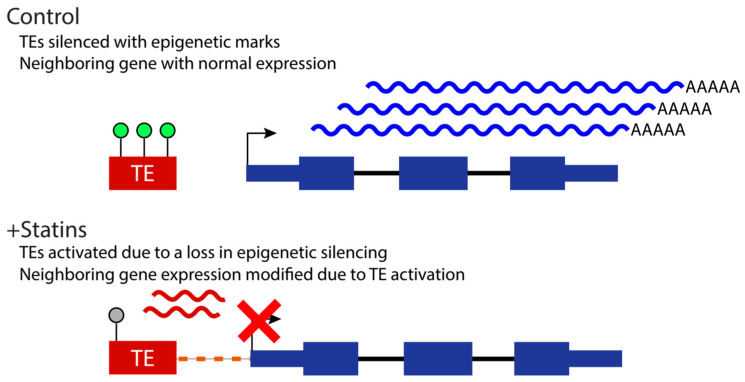
Proposed mechanism of TE activation upon statin treatment. Under normal conditions, TEs (red rectangle, “TE”) are epigenetically silenced (green circles above TE), and the expression of neighboring genes is uninterrupted (blue curly lines). Stress induced by statin treatment results in a loss of epigenetic silencing (grey circle above TE), which causes the TE to become activated (red curly lines), negatively influencing the expression of the neighboring gene.

Therefore, our results suggest that statin-dependent TEs expression regulates the expression levels of key components of skeletal muscle homeostasis signal pathways’ coding genes, representing a mechanism for statin-associated muscle symptoms and myopathy. Based on the “epi-transposon hypothesis,” and the body of knowledge indicating that stress can result in changes to TE expression, we propose a model in which statins cause modifications of the epigenetic marks on TEs, and this, in turn, can potentially impact the expression of neighboring genes (Figure 4). 

## 3. Materials and Methods

RNA-Seq datasets corresponding to control (no treatment and DMSO vehicle) and treated (simvastatin or rosuvastatin) samples were published by Grunwald et al. (2020), and are publicly available from the Sequence Read Archive (SRA) database (accession SRP126593). We obtained the sequencing libraries in FASTQ format using the SRA toolkit [41]. Mapping and quantification of reads per gene and TEs was performed using SQuIRE [28], which applies the Expectation-Maximization algorithm to quantify TE expression levels in a locus-specific manner. We first used “squire Fetch” to download the human genome hg38 version, and the respective gene and TE annotation files. “squire Fetch” calls STAR [42] to build a genome index for the subsequent read alignment step. Then, we processed the TE annotation file using “squire Clean” to obtain the annotation in BED format, which is required for the quantification step. Alignment to the STAR genome index was performed using “squire Map”, which sets the parameters --winAnchorMultimapNmax 100 and --outFilterMultimapNmax 100, in order to improve the mapping to TEs. After the alignment was done, quantification of reads per gene and TE was carried out using “squire Count.” The final output of this is a count matrix, which was then used for the differential expression analysis. 

Differential expression analysis among statin- (simvastatin, rosuvastatin) treated versus control (vehicle) samples were performed using DESeq2 [29]. A |log_2_(FC)| ≥ 1.5 threshold and adjusted *p*-value ≤ 0.05 were used as criteria for statistically significant expression levels. 

TEs locus-specific analysis was performed using BEDTools [43]. First, TEs overlapping with a gene sequence were classified as “Exon”, “Start codon”, “Stop codon” and “Intronic”, depending on which of the gene regions overlaps with it. TEs fully covered by either the exon, start or stop codons were discarded from the analysis, because in such cases, it is likely that the TEs belong to the gene reads rather than corresponding to *bona fide* TE expression. On the other hand, TEs that did not overlap with any of the aforementioned genic regions were classified as “Intergenic”. Based on this result, TEs were associated with genes if they had an overlap in terms of genomic locations, and in the case of intergenic TEs, they were associated to the closest gene.

Statistical associations between TEs and genes were obtained with TEffectR [30]. This tool applies a linear mathematical model, in which the response variable equals gene expression while the independent variable relates TE expression. We used, as input for TEffectR, the raw counts of differentially expressed TEs and the respective associated genes obtained in the previous locus-specific analysis. The statistical prediction allowed us to link changes in gene profile with TEs levels. Only gene–TE associations with *p*-value ≤ 0.05 were chosen. Then, the statistical correlation between gene–TE pairs was assessed using the ”cor” function of the R environment for statistical computing [44]. To examine the impact of TE expression onto gene regulation, only pairs with negative correlation were selected. To create a gene network for the selected TEs, the STRING database v11.0b [37] was used. All plots were obtained using ggplot2 [45].

## 4. Conclusions

Herein, we reported the first catalogue of TEs expressed upon statin treatment. Interestingly, there are differences in terms of amounts of TEs differentially expressed depending on the statin used, and as a consequence of this, in the amounts of TEs that can be statistically associated to genes. Moreover, the HMGCS1 gene has previously been reported to be increased upon statin treatment, and we found it to be associated with the AluSq2 TE in both simvastatin and rosuvastatin treatments. As reported previously, rosuvastatin seems to have fewer side effects than simvastatin. Thus, if TEs are responsible for these side effects or mediate them, our work adds another layer of evidence in elucidating the genetic cascade that occurs upon statin treatment. We argue that our findings will help the community interested in studying statin side effects, and propose that TEs can be a key target in ameliorating them. 

## Data Availability

Not applicable.

## Supplementary Materials

The following supporting information can be downloaded at: https://www.mdpi.com/article/10.3390/ijms24010244/s1.
