# Supplementary material for: Activation of Transposable Elements in Human Skeletal Muscle Fibers upon Statin Treatment
Source: Int J Mol Sci. 2022 Dec 23;24(1):244. doi: 10.3390/ijms24010244 (PMC9820482; doi:10.3390/ijms24010244)
Supplement: Supplementary file 1 [file ijms-24-00244-s001.zip › Supplementary File S2.pdf]

Supplementary File S2

Analysis of differentially regulated TEs across several criteria. The first cell in each table indicate the criteria, and then the proportions of TEs detected in that criteria for either Rosuvastatin or Simvastatin.

**Age distribution**	**Old**	**Young**
**Rosuvastatin**	85%	15%
**Simvastatin**	83%	16%

**Length**	**Full-length**	**Truncated**
**Rosuvastatin**	59%	41%
**Simvastatin**	38%	62%

**Relative position**	**Intergenic**	**Intragenic**
**Rosuvastatin**	11%	89%
**Simvastatin**	31%	69%

TE overlap with promoters

Only Simvastatin TEs were found to have an overlap with Promoters reported in The Eukaryotic Promoter Database

**TE**	**Promoter**	**Feature**	**TE expression**
chr11|9775467|9776058|MER21C:ERVL:LTR|+	noncoding	SBF2-AS1_2	Down-regulated
chr10|6737298|6737688|MLT1H:ERVL-MaLR:LTR|+	noncoding	LINP1_1	Up-regulated
chr12|70180099|70180628|MLT2B2:ERVL:LTR|+	gene	CNOT2_3	Down-regulated
chr14|102240324|102240748|HERVH-int:ERV1:LTR|+	gene	MOK_2	Down-regulated
chr4|114364773|114365325|MLT1E3:ERVL-MaLR:LTR|-	gene	ARSJ_2	Down-regulated
chr14|102508693|102508888|MIR1_Amn:MIR:SINE|-	gene	ANKRD9_2	Up-regulated
chr7|134527584|134527848|AluSx1:Alu:SINE|+	gene	AKR1B10_1	Up-regulated
chrX|38612501|38614189|HAL1:L1:LINE|-	gene	TSPAN7_4	Up-regulated

TE family distribution

Simvastatin

Family	Number
Alu	312
L1	276
MIR	173
L2	155
ERVL-MaLR	106
ERV1	66
hAT-Charlie	61
ERVL	47
TcMar-Tigger	34
CR1	20
ERVK	15
hAT-Tip100	15
SVA	9
tRNA-RTE	5
Gypsy?	4
RTE-X	4
TcMar-Mariner	4
Gypsy	3
hAT	3
hAT-Blackjack	3
5S-Deu-L2	1
ERV1?	1
ERVL?	1
LTR106_Mam	1
MER135	1
MULE-MuDR	1
MamRep605	1
RTE-BovB	1
TcMar-Tc2	1
hAT-Ac	1
hAT-Tip100?	1

Rosuvastatin

Family	Number
Alu	13
L1	6
MIR	5
ERVL	1
ERVL-MaLR	1
hAT-Charlie	1
