# Supplementary figures and images for: Activation of Transposable Elements in Human Skeletal Muscle Fibers upon Statin Treatment
Source: Int J Mol Sci. 2022 Dec 23;24(1):244. doi: 10.3390/ijms24010244 (PMC9820482; doi:10.3390/ijms24010244)

# A. Simvastatin

N=1326

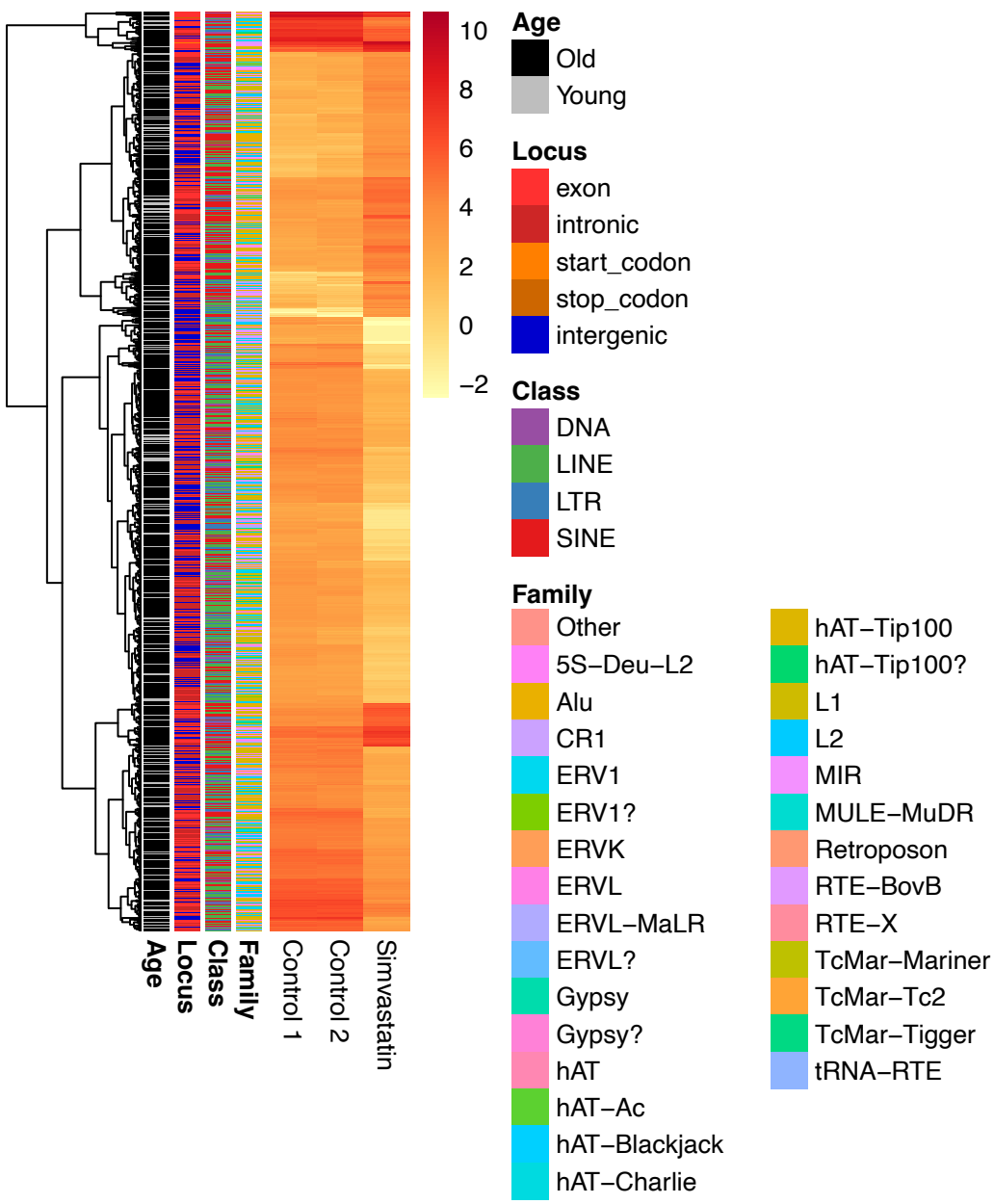

# B. Rosuvastatin

N=27

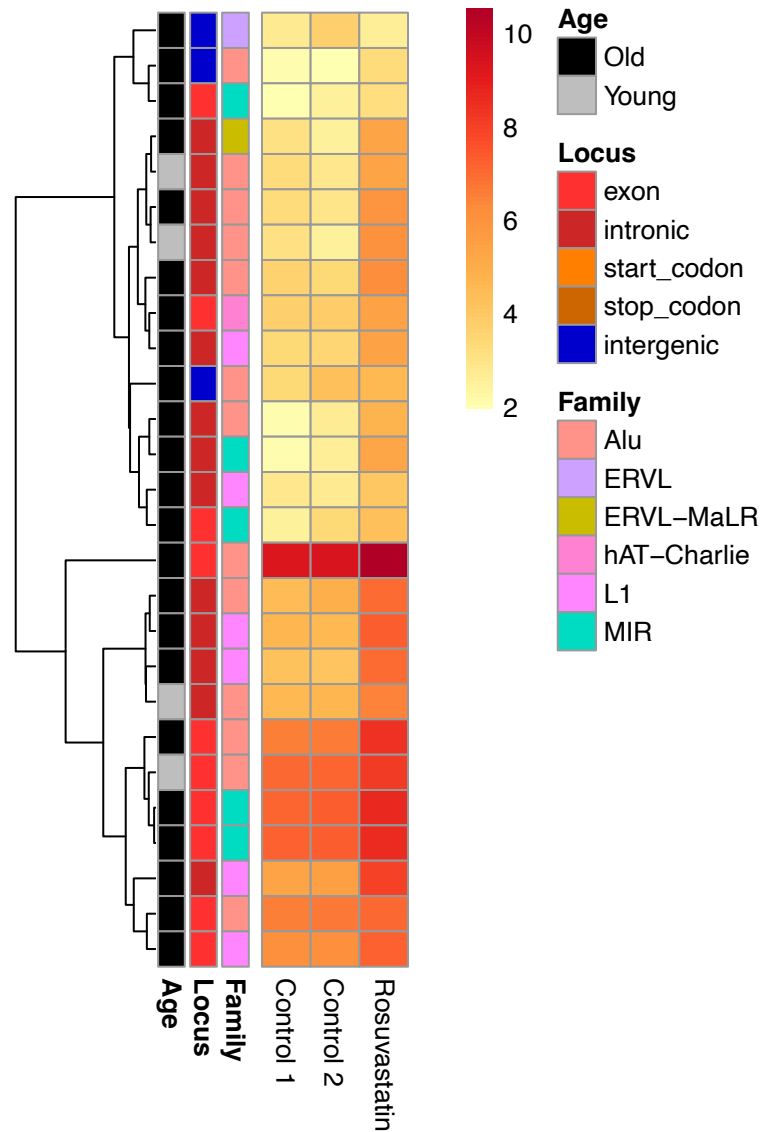

Supplement: Supplementary file 1 [file ijms-24-00244-s001.zip › Supplementary Figure S1.pdf]
